# 

**Published:** 1891-09

**Authors:** 


### CONTENTS.

ORIGINAL COMMUNICATIONS—

Laceration of the Anterior Vaginal Wall and Its Repair. By T. J. Watkins, M. D., Chicago, Ill. .... 385

Hysteria: Report of a Strange Case. By John Pope Stewart, M. D., Attalla, Ala. .... 392

Vomiting of Pregnancy—Its Etiology and Treatment. By F. Blume, M. D., Allegheny, Pa. .... 400

One Hundred Consecutive Cases of Skin Disease. VIII. By M. B. Hutchins, M. D., Atlanta, Ga. .... 410

SOCIETY REPORTS—

Gynecological and Obstetrical Society of Chicago .... 417

Allegheny Medical Society .... 420

EDITORIAL—

Some Remarks .... 436

SELECTIONS—

The Supposed Curative Effect of Operations *Per Se* .... 439

ITEMS .... 416, 435, 446, 447

BOOK REVIEWS .... 448

